# Posturography Approaches: An Insightful Window to Explore the Role of the Brain in Socio-Affective Processes

**DOI:** 10.3390/brainsci13111585

**Published:** 2023-11-12

**Authors:** Harold Mouras, Alexandre Vonesch, Karina Lebel, Guillaume Léonard, Thierry Lelard

**Affiliations:** 1 UR-UPJV 4559 LNFP Functional and Pathological Neurosciences Laboratory, Picardy Jules Verne University, 80054 Amiens, France; alexvonesch@hotmail.com; 2 Research Centre on Aging, CIUSSS de l’Estrie—CHUS, Sherbrooke, QC J1H 4C4, Canada; karina.lebel@usherbrooke.ca (K.L.); guillaume.leonard2@usherbrooke.ca (G.L.); 3 School of Rehabilitation, Faculty of Medicine and Health Sciences, Sherbrooke University, Sherbrooke, QC J1H 5N4, Canada; 4 UR-UPJV 3300 APERE Physiological Adaptation to Exercise and Exercise Rehabilitation, Picardy Jules Verne University, 80054 Amiens, France; thierry.lelard@u-picardie.fr

**Keywords:** stabilometry, socio-affective neurosciences, socio-emotional, embodiment, posture, motor correlates, neural correlates

## Abstract

A significant amount of research has highlighted the importance of a motor component in the brain’s processing of emotional, motivational and social information. Posturography has emerged as an interesting way to assess motor correlates associated with this process. In this review, we highlight recent results within the functional context of painful stimulus perception and discuss the interest in broadening the use of posturography to other motivational and societal functional contexts. Although characterized by significant feasibility, the single measurement of the COP’s anteroposterior displacement presents limitations for attesting approach–avoidance behavior towards a visual target. Here, we discuss a number of methodological avenues that could go some way towards overcoming these limitations.

## 1. The Historic Interaction between Emotion, Motivation and Motor Processes

The interaction between motor and emotional processes has been an object of interest for a long time, as revealed by the pioneering work of Darwin [1]. An ever-increasing number of scientific studies attest to the possibility of exploring the cerebral processing of socio-affective information by measuring both its central and peripheral correlates (see, for instance, [2,3]). Emotions are meant to prepare an organism to cope with major events; they produce a state of readiness for action that engages the whole individual and encourage the adoption of the most appropriate behaviors [4,5].

Numerous studies have demonstrated psychophysiological changes in response to emotional stimuli, accompanied by behavioral changes in terms of postural responses, vocal reactions and facial expressions (see [6] for review). Emotions confer a priority of control on states of readiness for action, claiming (not always successfully) priority in the control of behavior [5]. A stimulus from the environment contains emotional information that will trigger somatovisceral and motor responses and will prompt the individual to suspend action and act differently. Accordingly, the emotion–motor link is the cornerstone of a number of models and paradigms looking into emotional processing. Within the two-dimensional model of emotions, motivation is at the foreground of behavioral responses, with pleasant appetitive stimuli inducing approach-type responses and unpleasant defensive stimuli inducing withdrawal-type responses [7,8].

Among the advantages of posturography is its ability to investigate the spontaneous motor changes co-occurring during the processing of socio-affective information, making it a particularly interesting approach to study avoidance behaviors and other related phenomena. After all these years of research, we seize the opportunity to highlight the role and possible applications of posturography in the field, with an emphasis on studies that have measured the posturographic correlates related to the processing of socioemotional visual stimuli in different functional contexts. Methodological issues and recent advancements will also be discussed, with interesting prospects for the field made possible by these new technological developments, which now enable researchers to get out of the laboratory and take data analysis a step further.

## 2. Posturography as a Reflect of Approach–Avoidance Behavior

There are a multitude of posturographic parameters that allow the assessment of postural control (see [9]). Here, we report on the findings of studies that have been conducted to investigate approach–avoidance behavior in different emotional contexts, based on the anteroposterior position of the Center Of Pressure (COP).

When considering postural responses to visual stimulation, the anteroposterior COP displacement (COP-AP) is frequently used to highlight avoidance–approach processes. In many studies, the mean COP-AP is used to determine the extent to which a participant is leaning in the anterior or posterior direction during a trial, with higher values representing greater forward lean and lower values representing backward lean [10,11,12,13]. The mean COP position has been extensively used for research studying postural responses in postural threat situations and during emotional visual stimulation [14]. When considering postural responses to visual stimulation, COP-AP is generally used to highlight avoidance–approach processes. For example, in their task assessing postural interactions when faced with expressive faces, Lebert et al. [15] proposed that the anteroposterior position reflects action tendency behaviors. Ciria et al. [16] also used posturography to assess distance between the COP and the screen used to present the visual stimulus in order to estimate the avoidance–approach behavior toward aversive and appetitive stimuli. In postural threat tasks, the average location of the COP was referenced to the ankle joint and indicated how far an individual leaned away from the edge of the platform [13].

Overall, the COP parameter appears to be widely used to measure behavioral responses to emotions induced by visual stimulations or the manipulation of postural threats. An anterior modulation of the posture is generally interpreted as a reduction in the distance between the subject and the target (conscious or unconscious; i.e., an approach-type behavior), whereas a posterior modulation is interpreted as an increase in the distance between the subject and the target (conscious or unconscious; i.e., an avoidance-type behavior).

## 3. Preliminary Results on the Postural Correlates of Visual Emotional Stimuli Processing: On the Importance of Immersive Factors

In socioaffective neuroscience, posturographic tools have been used to capture postural modulations related to emotion within different functional contexts. One of these contexts for which different studies have produced an interesting body of data is the perception of painful stimuli. 

Ten years ago, a study introduced the use of posturography within the experimental model of empathy for pain [10]. This study demonstrated a differential modulation of postural control (indexed by the AP path) dependent on the valence of the stimuli. However, the results did not confirm the initial hypothesis of a withdrawal-type behavior in response to painful (i.e., negatively valenced) stimuli. This study shed light on the relationship between motor control and pain through spontaneous movement expressed by automatic postural responses for one of the first times. However, the researchers were unable to distinguish between the respective effect on postural modulation of mental simulation on one hand and of the valence of the stimuli on the other hand. Subsequent studies from the same research group incorporated both “passive vision” and “mental simulation” conditions to allow disentangling the effects of mental stimulation and pain on postural control [17,18]. In this last study, the researchers manipulated the instructions given to the participants to modulate their sense of involvement. They compared automatic motor responses when individuals were asked to imagine themselves in a non-painful, moderate and highly painful situation with a control condition (visualization of the scene without instructions). The results revealed that the participants’ degree of involvement has a significant impact on postural control modulation when dealing with emotional stimuli. Another important process that appeared in the results as a lever modulating the postural control associated with the processing of emotional stimuli was the subjects’ degree of involvement. These results could reflect a major role of mental simulation in the level of embodiment perceived. 

While an approach-type behavior was reported in the “passive observation” condition, a withdrawal response was measured in the “mental simulation observation” condition of a painful situation, suggesting that embodiment of a painful situation modulates motor responses and action tendencies. Of interest, these studies can be used to highlight potential dichotomies between subjective and objective avoidance measures. For example, in the same study by Beaumont et al. [18], a number of participants reported a high subjective sense of avoidance for pain images, while posturography revealed approach behaviors towards the painful stimuli. These apparently contradictory results could possibly be explained through the prism of the modulation of the cognitive processes (early, more “instinctive” processes vs. late, more cognitively controlled processes) that may participate in the modulation of postural control throughout the emotional stimulus perception period [19].

## 4. Postural Correlates of Affective Perception of the Environment

The information provided in this paper supports the interest in using posturography to study the peripheral and motor responses associated with the processing of emotional, motivational and social information. Interaction with our environment implies a fine articulation between motor and sensory responses, influenced both by the sensory modalities and the emotions involved [20]. The organization of human societies, the rules that govern them and the cognitive capacities specific to the human species make us particularly sensitive to problems that could be described as societal. At the forefront of these societal questions, it seems reasonable to think that appraisal processes and consequent behavioral responses articulate at least emotional, motivational and motor central components. Therefore, posturography may be an interesting tool to use to understand the processes at play and the behavioral responses involved. 

There is a large body of literature (not only scientific, but often more related to personal development or human resources) on the role of body language in social relations, in work contexts, etc. [21,22]. Given the scope of this work, we only consider here the field of static posturography as a tool for assessing the peripheral correlates (in relation to central processes) of the perception of a given emotional stimulus. The use of postural control measurements to assess cognitive, emotional and motivational processes in these contexts remains uncommon. A fortiori, the idea of using static posturography in “societal” contexts seems unlikely. However, it is interesting to mention original research that yielded interesting results using posturography in the context of environmental pollution. 

Recently, Beaumont et al. [18] explored the pleasure and subjective judgments of approach/avoidance evoked by stimuli related to the area of pollution. The main hypothesis of this study was that the perception of landscapes and scenes characterized by different degrees of pollution would induce different actions, notably via the modulation of emotional processes induced by the perception of these landscapes. The results enabled the researchers to establish a clear distinction between visual scenes of “clean” and “polluted” environments with respect to the subjective feelings induced by pleasure and approach–avoidance. This study was limited to the subjective evaluation of approach and avoidance behaviors based on participants’ verbal responses towards the scenes presented. Interestingly, Akounach et al. [23] attempted to overcome this limitation by adopting static posturography as an objective measure of this behavior. It appeared that the perception of polluted environments was associated with a lower tendency to approach, compared to clean environments, which could potentially be interpreted as avoidance reactions. Moreover, this differential pattern of postural modulation induced by pollution was correlated with ratings of feelings of pleasure and approach evoked by the images. Taken together, these results also support the idea that posturography could be used as a scientific method for the studying biological processes involved in societal contexts, mixing the induction of emotions and behavioral responses that may be related to the motor sphere. 

## 5. Methodological Issues in Socio-Affective Posturography

### The Combination of Posturography and Virtual Reality

As argued in a number of studies included in this article, one of the factors that seem to influence postural control during the processing of socio-affective information is the degree of involvement/immersion of the subject perceiving/processing the information. In different studies, the latter was experimentally manipulated through explicit instructions to project oneself into the represented scene. 

Another potential way of modulating this degree of involvement could be to vary the stimulation devices, for example, by using increasingly widespread virtual reality devices. In this sense, it would be particularly interesting to study the differential modulation of the subjects’ immersion depending on the stimulation modality (virtual reality (VR) device vs. screen). As the scientific literature is not very extensive on this subject, we can only provide herein information on two important questions prior to the use of virtual reality in socioaffective posturography: (i) the feasibility of the joint use of posturography and virtual reality helmets and (ii) the modulation of the level of involvement/immersion of subjects by virtual reality. These two questions are addressed successively in the following paragraphs. 

Assessment of the feasibility of using virtual reality in socio-affective posturography is herein based on studies conducted using healthy subjects (i) where the measurement of balance was conducted in a static and passive manner; (ii) that did not present a virtual environment that required interaction with virtual objects or exploration (also includes games) and where the images presented were intended to disrupt the volunteers’ balance; and (iii) that used the same stimuli delivered via the virtual reality headset or a conventional computer screen.

It is therefore particularly interesting to look at the results of three recent studies, whose main methodological characteristics, results and conclusions are summarized in Table 1.

The stimuli presented in the headset [24,26] were realistic reconstructions of the laboratories in which participants performed the experiment. The same participant then had to fix a target in the real environment and in the corresponding realistic virtual environment. In addition, Liang et al. [26] also presented a blank screen in the VR headset in which the participant had to imagine a target to stare at in front of him/her. In another study [25], the stimulus presented in the VR headset was a white image, without details, to match the white wall presented in the real environment, complemented at the periphery by two white blinds. Robert et al. [24] asked participants to stand with their arms at their sides, as steady as possible, and position their feet in a standardized manner. In the last study [26], several tasks were performed by the participants. For this, participants stood unsupported, their feet pointing 25° outward with a distance of 15 cm between their feet. For the visualization of the real environment, the participants were asked to position the VR headset on their foreheads so as not to feel the impact of the weight of the headset on their posture. However, in the Robert et al. study [24], participants were required to remove the headset to view the real environment. Imaizumi et al. [25], on the other hand, asked participants to position themselves comfortably on the force platform with both feet on the ground (side by side). Participants adhered to the following set of conditions in two experiments: (i) (Experiment 1) subjects wore or did not wear the VR headset and explored, respectively, the white wall or the white image without moving their heads; (ii) (Experiment 2) participants closed their eyes with or without the VR headset; (iii) participants opened their eyes while wearing the VR headset in which the white image was displayed or not (dark).

As reported in Table 1, most studies did not report a significant effect of the VR headset on postural control modulation. This supports the possible use of VR headsets on force platforms. Recently, this feasibility was confirmed by Nielsen et al. [27] in the specific context of socio-emotional neuroscience. The aim of this study was to assess the feasibility of eliciting visually evoked postural responses through VR devices and use them to examine the potential influence of virtual postural threat on the control of balance. The results demonstrated that visually evoked postural responses can be produced through VR devices with comparable evoked sway responses across experimental conditions (VR or not), with and without the presence of an elevated surface. Thus, the results of this study suggest that visual contributions to balance control are not strongly influenced by virtual postural threat. Taken together, these results confirm the feasibility of using VR devices in socio-affective contexts, while highlighting the limitations of such an approach.

Finally, it seems important to consider, for the joint use of VR and posturography, a few recommendations in the literature advising against including participants with cyberkinetosis, grouping a range of symptoms related to VR exposure. Other recommended exclusion criteria are (i) pregnant women; (ii) individuals with vestibular disorders; (iii) individuals with motion sickness; (iv) individuals with abnormalities of postural statics and/or dynamic balance with proprioception disorders; (v) individuals prone to oculomotor disorders and/or with ocular pathologies; (vi) individuals with migraine; and (vii) individuals with an anxious temperament. In addition, persons who are particularly sensitive to the light radiation emitted by the devices should be excluded, such as (i) persons with sleep disorders and (ii) persons with photosensitive epilepsy.

## 6. Methodological Challenges 

### 6.1. Development of Motion Capture Tools

Kinetic and kinematic tools such as optoelectronic motion capture systems or force plates are widely used in motion analysis laboratories for biomechanical analysis of gait and postural control. Though accurate, these systems impose constraints on the environment, are costly and require technological expertise to operate and to analyze the data [28]. Moreover, when assessing emotional response, it has been reported that experiments conducted with artificial, controlled laboratory parameters do not seem to provide a complete understanding of the complexity of the processes involved in the modulation of motor responses by psycho-emotional factors [29]. Recent advances in technological developments, including wearable sensors and AI-based software to analyze posture, offer new potential setups to assess emotional response, which may overcome some of these limitations. This section provides a perspective on current and foreseen kinetic and kinematic systems to assess emotional response, with a focus on the potential for the development of an ecological experimental approach based on a reliable, easy-to-use movement analysis tool. 

### 6.2. Force Plates

As discussed previously, the classical approach in posturography is based on the analysis of the COP measured using force plates. The collection of COP displacement measurements is simple and convenient, which explains why force-plate-based posturography is the most commonly used approach in clinical studies [30]. The concept assessed through this approach refers to postural sway, meaning the displacement of a person’s enter Of Mass (COM) above their base of support [31,32]. Indeed, force plates under the person’s feet measure the point of application of the vector of force produced by gravity acting on the COM [33]. Thus, COP and COM are related concepts, but direct comparison requires the use of a model, the most popular one being an inverted pendulum, though more complex modelling approaches are arising in the literature [31]. Though useful, the collected information remains partial as no detailed information on the kinematics is available [29]. A biomechanical model used to describe postural changes based on the unique collection of the center of pressure must thus be used with caution, considering the number of degrees of freedom involved in the human body. Kinematic can supplement kinetic data to create a more reliable characterization of motor responses to emotional stimulation.

### 6.3. Optoelectronic Motion Capture Systems

Camera-based optoelectronic motion capture systems are recognized as a gold standard to capture kinematic data [28]. Traditional 3D-based systems are made of a set of infrared cameras carefully positioned to cover an volume of interest [28]. Calibration procedures allow us to determine the relative position and orientation of all cameras and to anchor this information into a reference frame based on the lab. Markers are then affixed onto specific anatomical reference points on the participant. Through a triangulation process, the 3D position of each marker is determined by the system. An anatomical model can then be applied to link the different markers to segments of interest, allowing us to capture kinematics. Analysis of kinematic variations over time provides information on postural control. These systems can thus be used to estimate the COM position and also to analyze the general postural variations during perturbations. Regardless of its precision, this type of system is rarely used in emotional response assessment due to the complexity of the setup involved, its availability or the cost involved.

### 6.4. Inertial Systems

Over the last two decades, researchers have shown a growing interest in inertial sensors for motion capture. Indeed, advances in microelectronics have allowed us to develop compact portable sensors at a relatively low cost. Inertial sensors can sense motion, based on the physical laws of motion. This type of sensor generally includes accelerometers and gyroscopes. Accelerometers measure the total free-body linear acceleration sensed by the device. In other words, they measure the combined gravitational force and linear acceleration. As a result, accelerometers are used as inclinometers in static conditions. If the accelerometer is aligned with a segment, knowledge of the gravitational acceleration vector will allow computation of the segment orientation in 2D, also referred to as 2D static posture [34]. When movement is incorporated into the measure, two different challenges arise. The first challenge relates to the necessity of dissociating gravitational acceleration from the acceleration associated with motion, while the second issue arises from the impact of rotational motion on linear acceleration. Indeed, inertial sensors must be carefully affixed onto a body segment to minimize soft tissue artifacts in the measurement. Consequently, sensors are attached away from joints’ center of rotation. The captured linear acceleration is thus partly corrupted by the rotational movement of the segment [35]. Depending on the accelerometer’s type of use, these issues may have to be considered within the data analysis. Static postural sway has been successfully appraised using accelerometers mainly positioned on the lower back, without concern for the above-raised issues [36,37,38]. Indeed, these conditions can be considered quasi-static, meaning with limited motion. Linear acceleration due to motion is thus negligible compared to gravity. Yet, as mentioned by Ghislieri et al. [37], sensor calibration and alignment may have an impact on the quality of the measures. In emotion posturography, the motion introduced by the perturbation may impact the quality of the COM estimate, especially if one relies on position-based variables. 

These days, most accelerometers and gyroscopes are packaged into inertial measurement units (IMUs). As such, it is possible to make good use of the complementary information provided by the different sensors to improve the quality of our measurements. Indeed, some IMUs incorporate fusion filters to estimate the sensor’s orientation in space [39,40,41,42]. Knowledge of this orientation then has the potential to (i) better inform on COM inclination, and (ii) “clean up” the data, decoupling rotational and linear movement information. Yet, the quality of the estimation filter depends directly upon the calibration of various parameters [41,43]. For example, complementary filters estimate orientation from accelerometers in static conditions, moving to integration of angular velocity provided by the gyroscope when movement is implied. The quality of orientation estimation will thus vary depending on the characteristics determined to switch between the modes and its fit with the actual context that the IMU is used in. The EKF (Extended Kalman Filter) belongs to the Kalman Filter (KF) family, which estimates the missing states, based on the equation of motions and actual measurements. The general principles of the KF rely on the right balance between theoretical propagation of the states using previous information, equations of motion, and its actual measurements. Accuracy of estimation thus highly relies on the tuning of this balance. The traditional KF is, however, limited to linear equations. The EKF is an extension of the traditional KF that applies to nonlinear equations of motion, as long as these equations are linearized about a meaningful point of operation. When the motion assessed is close to the chosen point of operation for which the EKF was optimized, estimated states will be as good as those reported in the manufacturer’s specifications.

Body kinematics can also be deduced from IMUs, using information provided by multiple units, and an anatomical model. For example, Germanotta et al. [38] used seven IMUs to reconstruct full-body kinematics. This full-body kinematics was then used to estimate the COM and assess COM sway against an optoelectronic gold standard with 15 healthy participants. The authors revealed an excellent correlation for AP sway, ML sway, 95% sway area and mean sway velocity in free sway conditions. Inertial sensors therefore appear to have an interesting potential for emotional response assessment. However, not all inertial systems have been created equally and their validity for the specific context of use should therefore be carefully verified prior to use in a clinical context [37]. 

### 6.5. RGB Cameras

Regular RGB cameras have been used for a long time to capture a visual on a person’s posture. Yet, the two-dimensional images used limit objective assessment of posture with cameras. To assess posture changes from 2D images, cameras can be positioned perpendicular to the plane of motion of interest. Segments can then be identified manually and changes in posture estimated. Yet, accuracy of the measure directly depends upon the precision of the camera’s orientation and on appropriate scaling, due to the distance between the camera and the person. To overcome these limitations, two different approaches arose [44]. First, some cameras now offer complementary depth information. The so-called RGB-D cameras allow us to automatically retrieve 3D information. However, it should be noted that most consumer-grade RGB-D cameras have different specifications when it comes to depth, compared to the basic RGB information. For example, the Intel Realsense D435 offers an RGB video with a resolution of 1920 × 1080, while the depth information for the same camera has a resolution of up to 1280 × 720. The quality of the estimation may therefore be affected. Nevertheless, the potential of RGB-D for posturography appears real. Recently, Bertram et al. [45] determined an excellent correlation between lower-trunk measurements assessed with the Azure Kinect in comparison to an optoelectronic gold standard with 30 healthy adults. The study revealed a strong correlation for lower-trunk position measurement in both anteroposterior (r = 0.94) and mediolateral (r = 0.75) directions during standing tasks. The other potential solution to improve regular RGB estimation relies on the use of algorithms based on artificial intelligence to autonomously detect a person’s skeleton from 2D images. Amongst these algorithms, OpenPose and MediaPipe appear the most popular. A recent study by Lafayette et al. [46] compared the accuracy of joint angles assessed from regular RGB videos processed with MediaPipe and joints assessed from RGB-D videos, both against an optoelectronic gold standard with six healthy adults performing upper and lower limb movements. This study reports a slight advantage for the RGB + MediaPipe approach over the RGB-D direct computation. Indeed, the MediaPipe algorithm allowed an average accuracy of 9° and an excellent correlation (r = 0.86) with the reference system. Yet, this study considered important movements of upper and lower limbs, which is not the case in posturography. To the authors’ knowledge, very little of the literature has properly validated the use of these algorithms in the context of posturography. As such, specific guidelines for the use of this type of approach for posturography should be determined to ensure a reliable assessment.

Table 2 summarizes the strengths and weaknesses identified for the motion capture approaches discussed above, in relation to their use in posturography. Traditional laboratory approaches such as force plates and optoelectronic motion capture systems provide accurate biomechanical analysis, though they appear less appropriate for ecological setups. On the other hand, inertial systems offer a portable solution with some interesting potential, though the specificity of the chosen system should be validated prior to use. Finally, the relatively recent developments of AI algorithms for autonomous posture detection appear to offer new possibilities for RGB-camera-based assessment, though the accuracy and reliability of this approach remain to be validated. 

### 6.6. Methodological Challenges: The Place of New Analytical Methods

When considering research on the use of posturography in social–emotional neuroscience, data analysis typically focuses on comparisons of means of posturographic indices across experimental conditions. 

Interestingly, a number of publications highlight the value of alternative methods of posturographic data analysis. When analyzing postural data, the nonlinear methods give complementary information to the traditional methods (see [47] for review). For example, multiscale entropy has made it possible to address the complexity of postural control (in healthy subjects, see [48]). In general, nonlinear measures (sample entropy, fractal dimension, Lyapunov exponent used as nonlinear measures) have shown their potential in the exploration of postural control and the dynamics of the trajectory of the body pressure center in different functional contexts. To the best of our knowledge, only a few studies have made use of these non-linear calculation methods to explore postural responses to emotional stimuli. However, they seem to offer interesting perspectives [47].

In the context of social–emotional neuroscience, these analyses and measures would be of particular interest to consider in further exploring hypotheses and questions, as well as in addressing the issue of the temporal dynamics of postural response [49].

### 6.7. Perspective Linking Posturographic and Central Neural Measurements

This review aims to illustrate how posturographic approaches can be a valuable tool for exploring the role of the brain in the socio-affective process. An interesting and central question is the joint use of posturography and simultaneous neural activity collection methods. One of the important limitations of studies on the posturographic correlates of behavior is the lack of information on the nature of the cognitive processes presiding over the recorded postural modulation. This makes interpretations of the measured effects relatively difficult. A potential solution to this limitation would be to simultaneously collect posturographic and neural correlates in the same experimental time frame. Among the available neuroimaging techniques, electroencephalography seems to be more compatible with a classical stabilometric experimental setting. Indeed, when looking at the scientific literature, a certain number of studies have jointly measured posturography and electroencephalography [49,50]. These studies document the feasibility of the concurrent use of posturography and electroencephalography to explore the neural signature of postural control.

More recently, electroencephalography has been recorded concurrently with passive stabilometry in response to socio-emotional stimuli presentation [50]. On the EEG side, classical findings have been documented with results of decreased alpha and increased gamma power over posterior areas in response to unpleasant compared to pleasant pictures (and compared to neutral pictures for gamma power). Although these studies failed to show a significant correlation between posturographic markers (characterizing a freezing reaction [51], for example) and neural markers, the authors of the study are confident about future studies and attribute this lack of results to methodological limitations. Technological advancements in the exploration of human movement lead us to believe that it will be possible to synchronously study behavioral and physiological responses in ecological settings.

## 7. Conclusions

This review provides an overview of the state of knowledge surrounding the interaction between postural responses and socioemotional processes, highlighting as a canonical example result obtained in a very interesting functional context, i.e., painful stimulus perception. Although an interesting functional context, it seems also interesting to broaden the range of functional contexts in which posturography is used with other motivational contexts (alcohol and erotic incentives; [52,53]) or more societal ones (pollution perception; [23]). The over-representation of studies using COP displacement compared to other biomechanical models is likely due to the simplicity and ease of use of this type of measurement. Nevertheless, this metric has limitations, as it does not adequately account for the complexity of the movements involved in postural control. The recent development of markerless kinematic measurement devices suggests the development of new applications, allowing for a better understanding of human behavior in socioemotional contexts.

## Figures and Tables

**Table 1:** Recent studies on VR and posturography.

Reference	Number of Participants	Age	Stimuli	Main Results on Static Postural Modulation	Conclusion
Robert et al., 2016 [23]	14 (9 M–5 W)	26.1 ± 3.1	VR: 360° picture of the lab Real: Target in the lab	No significant difference between the virtual and physical environment	Display of a virtual environment from a photo can be used during static balance in healthy adults
Imaizumi et al., 2020 [24]	E1: 44 (30 M–14 W) E2: 24 (7 M–8 W)	E1: 22 ± 2 E2: 24 ± 3	VR: White picture Real: White wall	E1: Increased COP displacement when viewing the stimulus in a VR headset compared to the condition without the headset E2: No significant difference between eyes closed with VR headset and without; no significant difference for eyes-open conditions with VR headset with or without stimulus	VR headsets disrupt the balance of young adults during a standing postural task with eyes open. These effects disappear with eyes closed or without stimulus. Indicates the importance of reliable visual inputs in the virtual environment during a standing postural task
Liang et al., 2021 [25]	34 (12 M–22 W)	26.5 ± 6.3	VR: Picture of the lab and white screen Real: Target in the lab	No significant difference between the realistic virtual environment and the physical environment (standardized position)	Visual balance dependence is similar between viewing a target in scenes in the VR headset and in the physical environment, except for difficult tasks

**Table 2:** Current and foreseen systems for motion capture in the specific context of posturography.

System	Measurement	Strengths	Weaknesses	Ecological Potential
Force plate/posturographic platform	COP	Accuracy	Lab setup mainly Requires a model to estimate COM	Limited
Optoelectronic motion capture	Full-body kinematics (posture)	Accuracy	Time to setup Complexity (setup, collect, analyze) High cost Potential obstructions	Very low
Inertial systems	Segments’ orientation	Portability Relatively low cost	Trade-offs between cost and fidelity/complexity in analysis depending on the system used	High
RGB cameras	Manual detection of changes between frames	Portable Accessible	Manual intervention Relative position of the camera to the person may affect accuracy	Moderate
RGB cameras + AI	General posture	Simplicity Low cost	Accuracy may be affected by environmental parameters (e.g., luminosity), by the required scaling to obtain usable data for posture analysis and by the angle between the participant and the camera	High

## References

[1] Darwin C., Prodger P. (1998). The Expression of the Emotions in Man and Animals.

[2] Cromwell H.C., Papadelis C. (2022). Mapping the brain basis of feelings, emotions and much more: A special issue focused on ‘The Human Affectome’. Neurosci. Biobehav. Rev..

[3] Grimshaw G.M., Philipp M.C. (2021). Bodies in mind: Using peripheral psychophysiology to probe emotional and social processes. J. R. Soc. N. Z..

[4] Scherer K.R. (2009). Emotions are emergent processes: They require a dynamic computational architecture. Philos. Trans. R. Soc. B Biol. Sci..

[5] Frijda N.H. (2010). Impulsive action and motivation. Biol. Psychol..

[6] Branco D., Gonçalves Ó.F., Badia S.B.I. (2023). A Systematic Review of International Affective Picture System (IAPS) around the World. Sensors.

[7] Bradley M.M., Codispoti M., Cuthbert B.N., Lang P.J. (2001). Emotion and motivation I: Defensive and appetitive reactions in picture processing. Emotion.

[8] Horslen B.C., Carpenter M.G. (2011). Arousal, valence and their relative effects on postural control. Exp. Brain Res..

[9] Paillard T., Noé F. (2015). Techniques and methods for testing the postural function in healthy and pathological subjects. BioMed Res. Int..

[10] Lelard T., Montalan B., Morel M.F., Krystkowiak P., Ahmaidi S., Godefroy O., Mouras H. (2013). Postural correlates with panful situations. Front. Hum. Neurosci..

[11] Stins J.F., Beek P.J. (2007). Effects of affective picture viewing on postural control. BMC Neurosci..

[12] Stins J.F., Roerdink M., Beek P.J. (2011). To freeze or not to freeze ? Affective and cognitive perturbations have markedly different effects on postural control. Hum. Mov. Sci..

[13] Zaback M., Cleworth T.W., Carpenter M.G., Adkin A.L. (2015). Personality traits and individual differences predict threat-induced changes in postural control. Hum. Mov. Sci..

[14] Lelard T., Stins J., Mouras H. (2019). Postural responses to emotional visual stimuli. Clin. Neurophysiol..

[15] Lebert A., Chaby L., Garnot C., Vergilino-Perez D. (2020). The impact of emotional videos and emotional static faces on postural control through a personality trait approach. Exp. Brain Res..

[16] Ciria L.F., Muñoz M.A., Gea J., Peña N., Miranda J.G.V., Montoya P., Vila J. (2017). Head movement measurement: An alternative method for posturography studies. Gait Posture.

[17] Lelard T., Godefroy O., Ahmaidi S., Krystkowiak P., Mouras H. (2017). Mental simulation of painful situations has an impact on posture and psychophysiological parameters. Front. Psychol..

[18] Beaumont A., Granon S., Godefroy O., Lelard T., Mouras H. (2021). Postural correlates of painful stimuli exposure: Impact of mental simulation processes and pain-level of the stimuli. Exp. Brain Res..

[19] LeDoux J.E. (1998). The Emotional Brain: The Mysterious Underpinnings of Emotional Life.

[20] Riečanský I., Lamm C. (2019). The role of sensorimotor processes in pain empathy. Brain Topogr..

[21] De Gelder B. (2006). Towards the neurobiology of emotional body language. Nat. Rev. Neurosci..

[22] White J., Gardner J. (2013). The Classroom x-Factor: The Power of Body Language and Non-Verbal Communication in Teaching.

[23] Akounach M., Lelard T., Beaumont A., Granon S., Mouras H. (2022). Postural correlates of pollution perception. Brain Sci..

[24] Robert M.T., Ballaz L., Lemay M. (2016). The effect of viewing a virtual environment through a head-mounted display on balance. Gait Posture.

[25] Imaizumi L.F.I., Polastri P.F., Penedo T., Vieira L.H.P., Simieli L., Navega F.R.F., de Mello Monteiro C.B., Rodrigues S.T., Barbieri F.A. (2020). Virtual reality head-mounted goggles increase the body sway of young adults during standing posture. Neurosci. Lett..

[26] Liang H.W., Chi S.Y., Chen B.Y., Li Y.H., Tai T.L., Hwang Y.H. (2021). The effects of visual backgrounds in the virtual environments on the postural stability of standing. IEEE Trans. Neural Syst. Rehabil. Eng..

[27] Nielsen E.I., Cleworth T.W., Carpenter M.G. (2022). Exploring emotional-modulation of visually evoked postural responses through virtual reality. Neurosci. Lett..

[28] Zhou H., Hu H. (2008). Human motion tracking for rehabilitation—A survey. Biomed. Signal Process. Control.

[29] Troncoso A., Soto V., Gomila A., Martínez-Pernía D. (2023). Moving beyond the lab: Investigating empathy through the Empirical 5E approach. Front. Psychol..

[30] Crétual A. (2015). Which biomechanical models are currently used in standing posture analysis?. Neurophysiol. Clin./Clin. Neurophysiol..

[31] Richmond S.B., Fling B.W., Lee H., Peterson D.S. (2021). The assessment of center of mass and center of pressure during quiet stance: Current applications and future directions. J. Biomech..

[32] Winter D.A. (1995). Human balance and posture control during standing and walking. Gait Posture.

[33] Hallett M., DelRosso L.M., Elble R., Ferri R., Horak F.B., Lehericy S., Mancini M., Matsuhashi M., Matsumoto R., Muthuraman M. (2021). Evaluation of movement and brain activity. Clin. Neurophysiol..

[34] Buldt A.K., Murley G.S., Butterworth P., Levinger P., Menz H.B., Landorf K.B. (2013). The relationship between foot posture and lower limb kinematics during walking: A systematic review. Gait Posture.

[35] Allmond J., Hakhamaneshi M., Wilson D., Kutter B. (2014). Advances in Measuring Rotation with MEMS Accelerometers.

[36] Neville C., Ludlow C., Rieger B. (2015). Measuring postural stability with an inertial sensor: Validity and sensitivity. Med. Devices Evid. Res..

[37] Ghislieri M., Gastaldi L., Pastorelli S., Tadano S., Agostini V. (2019). Wearable inertial sensors to assess standing balance: A systematic review. Sensors.

[38] Germanotta M., Mileti I., Conforti I., Del Prete Z., Aprile I., Palermo E. (2021). Estimation of human center of mass position through the inertial sensors-based methods in postural tasks: An accuracy evaluation. Sensors.

[39] McGinnis R.S., Cain S.M., Davidson S.P., Vitali R.V., McLean S.G., Perkins N.C. (2014). Validation of complementary filter based IMU data fusion for tracking torso angle and rifle orientation. Proceedings of the ASME International Mechanical Engineering Congress and Exposition.

[40] Zhao H., Wang Z. (2011). Motion measurement using inertial sensors, ultrasonic sensors, and magnetometers with extended kalman filter for data fusion. IEEE Sens. J..

[41] Caruso M., Sabatini A.M., Laidig D., Seel T., Knaflitz M., Della Croce U., Cereatti A. (2021). Analysis of the accuracy of ten algorithms for orientation estimation using inertial and magnetic sensing under optimal conditions: One size does not fit all. Sensors.

[42] Nazarahari M., Rouhani H. (2021). Sensor fusion algorithms for orientation tracking via magnetic and inertial measurement units: An experimental comparison survey. Inf. Fusion.

[43] Tedaldi D., Pretto A., Menegatti E. (2014). A robust and easy to implement method for IMU calibration without external equipments. Proceedings of the 2014 IEEE International Conference on Robotics and Automation (ICRA).

[44] Marusic A., Nguyen S.M., Tapus A. Evaluating Kinect, OpenPose and BlazePose for Human Body Movement Analysis on a Low Back Pain Physical Rehabilitation Dataset. Proceedings of the Companion of the 2023 ACM/IEEE International Conference on Human-Robot Interaction.

[45] Bertram J., Krüger T., Röhling H.M., Jelusic A., Mansow-Model S., Schniepp R., Wuehr M., Otte K. (2023). Accuracy and repeatability of the Microsoft Azure Kinect for clinical measurement of motor function. PLoS ONE.

[46] Lafayette T.B.D.G., Kunst V.H.D.L., Melo P.V.D.S., Guedes P.D.O., Teixeira J.M.X.N., Vasconcelos C.R.D., Teichrieb V., Da Gama A.E.F. (2022). Validation of Angle Estimation Based on Body Tracking Data from RGB-D and RGB Cameras for Biomechanical Assessment. Sensors.

[47] Kędziorek J., Błażkiewicz M. (2020). Nonlinear Measures to Evaluate Upright Postural Stability: A Systematic Review. Entropy.

[48] Busa M.A., van Emmerik R.E. (2016). Multiscale entropy: A tool for understanding the complexity of postural control. J. Sport Health Sci..

[49] Barollo F., Friðriksdóttir R., Edmunds K.J., Karlsson G.H., Svansson H.Á., Hassan M., Gargiulo P. (2020). Postural control adaptation and habituation during vibratory proprioceptive stimulation: An HD-EEG investigation of cortical recruitment and kinematics. IEEE Trans. Neural Syst. Rehabil. Eng..

[50] Stokkermans M., Solis-Escalante T., Cohen M.X., Weerdesteyn V. (2023). Midfrontal theta dynamics index the monitoring of postural stability. Cereb. Cortex.

[51] Luther L., Horschig J.M., van Peer J.M., Roelofs K., Jensen O., Hagenaars M.A. (2023). Oscillatory brain responses to emotional stimuli are effects related to events rather than states. Front. Hum. Neurosci..

[52] Mouras H., Lelard T., Ahamaidi S., Godefroy O., Krystkowiak P. (2015). Freezing behavior as a response to sexual visual stimuli as demonstrated by posturography. PLoS ONE.

[53] Noël X., Dubuson M., Rougier M., Lelard T., Mouras H., Kornreich C., Wyckmans F., Pereira M., Chatard A., Némat J. (2021). Distinct whole-body movements in response to alcohol and sexual content in alcohol use disorder. Alcohol. Clin. Exp. Res..

